# Tunable Goos-Hänchen Shift Surface Plasmon Resonance Sensor Based on Graphene-hBN Heterostructure

**DOI:** 10.3390/bios11060201

**Published:** 2021-06-21

**Authors:** Zihao Liu, Fangyuan Lu, Leyong Jiang, Wei Lin, Zhiwei Zheng

**Affiliations:** School of Physics and Electronics, Hunan Normal University, Changsha 410081, China; liuzihao0514@hunnu.edu.cn (Z.L.); 201830133040@smail.hunnu.edu.cn (F.L.); jiangly28@hunnu.edu.cn (L.J.); linweihbhg@hunnu.edu.cn (W.L.)

**Keywords:** Goos-Hänchen (GH) shift, graphene, sensor

## Abstract

In this paper, a bimetallic sensor based on graphene-hexagonal boron nitride (hBN) heterostructure is theoretically studied. The sensitivity of the sensor can be improved by enhancing the Goos–Hänchen (GH) shift in the infrared band. The theoretical results show that adjusting the Fermi level, the number of graphene layers and the thickness of hBN, a GH shift of 182.09 *λ* can be obtained. Moreover, sensitivity of 2.02 × 10^5^ *λ/RIU* can be achieved with monolayer graphene, the thickness of gold layer is 20 nm, silver layer is 15 nm, and the hBN thickness of 492 nm. This heterogeneous infrared sensor has the advantages of high sensitivity and strong stability. The research results will provide a theoretical basis for the design of a new high-sensitivity infrared band sensor.

## 1. Introduction

Goos–Hänchen (GH) shift refers to the slight lateral deviation of the actual reflected beam relative to the geometrical optics predicted beam when the beam is reflected at the interface between the media. Since it was demonstrated by Goos and Hänchen in 1947 [1], GH shift has been widely studied in optical waveguide switch [2], narrow band optical filter [3], high sensitivity temperature sensor [4] and other fields. However, usually, the GH shift of the optical band is only in the order of wavelength, which makes it difficult to be measured in experiments, which hinders its practical application to a certain extent. In the past decade, the rapid development of nano-machining technology has set off a research boom of various new types of artificial microstructure materials. Thus, a diverse variety of microstructures has been designed by researchers to amplify GH shift. For example, negative refractive index materials [5], weak absorption medium dielectric slab [6], symmetrical metal-cladding optical waveguide [7] and periodic structure [8], photonic crystal [9], etc. However, after the internal parameters of these traditional structures are fixed, it is difficult to achieve passive regulation. This makes flexible regulation of GH displacement based on new semiconductor materials combined with traditional structures become a new research direction.

In recent years, two-dimensional materials represented by graphene and molybdenum disulfide (MoS_2_) have excellent optoelectronic characteristics such as high electron mobility and adjustable external field [10,11,12]. Jiang et al. studied the GH shift of the reflected light beam on the graphene and realized the positive and negative conversion of the GH shift under the action of electronic control [13]. Tang et al. achieved a tunable GH shift in the terahertz band by utilizing magneto-optical effects in graphene [14]. It can be found from many cases of regulating GH shift that the material characteristics and structural parameters can have a significant impact on GH shift, so it is a good method to realize sensing performance by monitoring GH shift signal. Based on this idea, Zhou et al. designed a refractive index sensor with adjustable sensitivity coefficient by precisely controlling the GH shift in the graphene-substrate system at the 1550 nm optical communication band. By tuning the Fermi energy, the sensitivity coefficient can reach ±1 × 10^6^ [15]. Also, You et al. proposed that based on the graphene-MoS_2_ heterostructure, under the action of two MoS_2_ layers and three layers of graphene at the 632.8 nm band, the maximum GH shift is obtained and the optimum sensitivity of 5.545 × 10^5^ *λ* is achieved [16]. Therefore, the application of two-dimensional materials to the sensor in GH shift interrogation mode can not only obtain large GH shifts, but also realize the flexible adjustment of sensitivity coefficient.

In addition, improving sensor sensitivity has always been the goal of researchers. Traditional types of sensors usually use Au and Ag to improve sensor sensitivity [17]. For example, at 633 nm wavelength, Shushama et al. designed a refractive index sensor in angular interrogation mode by combining a two-dimensional material with gold. The proposed sensor has a maximum sensitivity of 210°/*RIU* [18]. Fouad et al. achieved a high sensitivity of 280°/*RIU* using an Ag–BaTiO_3_ sensor at 632.8 nm [19]. However, these sensors also have some shortcomings. On the one hand, due to the poor binding ability of Au with biomolecules, it is difficult to improve the sensitivity of the sensors. On the other hand, although pure silver can provide a higher sensitivity, the pure silver exposed in the air oxidizes easily and has poor chemical stability [20]. So, researchers are considering combining gold and silver as bimetallic sensors to improve the chemical stability and sensitivity of the device. Although higher sensitivity can be provided by pure silver, it is easy to oxidize and has poor chemical stability. Therefore, the bimetallic sensors composed of gold and silver are used to improve the chemical stability and sensitivity of the devices. Since its more useful optical properties than pure gold or silver, sensor based on bimetal have also attracted a lot of attention [21,22]. Such as Liu et al. reported a novel biosensor in angular interrogation mode, which has an Ag/Au/BaTiO_3_/graphene bimetallic configuration at the wavelength of 633 nm. Sensitivities as high as 294°/*RIU* were obtained by optimizing the parameters [23]. The success of these cases also provides a way to improve the sensitivity of the sensor in GH shift interrogation mode. However, in general, researchers mostly consider improving the sensitivity of the sensor in the optical band, and the infrared band is an important band of solar radiation, so it is more valuable to apply the composite structure to the infrared band. It is worth mentioning that hexagonal boron nitride (hBN), as a natural hyperbolic material, is often used as a substrate material and a dielectric material for high-performance devices in the infrared band. Due to its unique phonon characteristics, the graphene-hBN heterostructure has exhibited many excellent optical properties in the infrared band [24]. For example, in the infrared band, a graphene-hBN heterostructure is used to improve the reflected group delay [25], achieve perfect absorption [26], and enhance near-field thermal radiation [27].

Inspired by the above discussion, it has been shown that a bimetal composite structure based on graphene-hBN can achieve a large GH shift and high-sensitivity sensors. A giant GH shift was obtained by optimizing the Fermi energy, the number of graphene layers, and the hBN thickness. In addition, our structure can also be used as a sensitivity-adjustable refractive index sensor based on GH shift modulation to obtain the best sensitivity up to 2.02 × 10^5^ *λ/RIU*.

## 2. Theoretical Model

**Figure 1:**
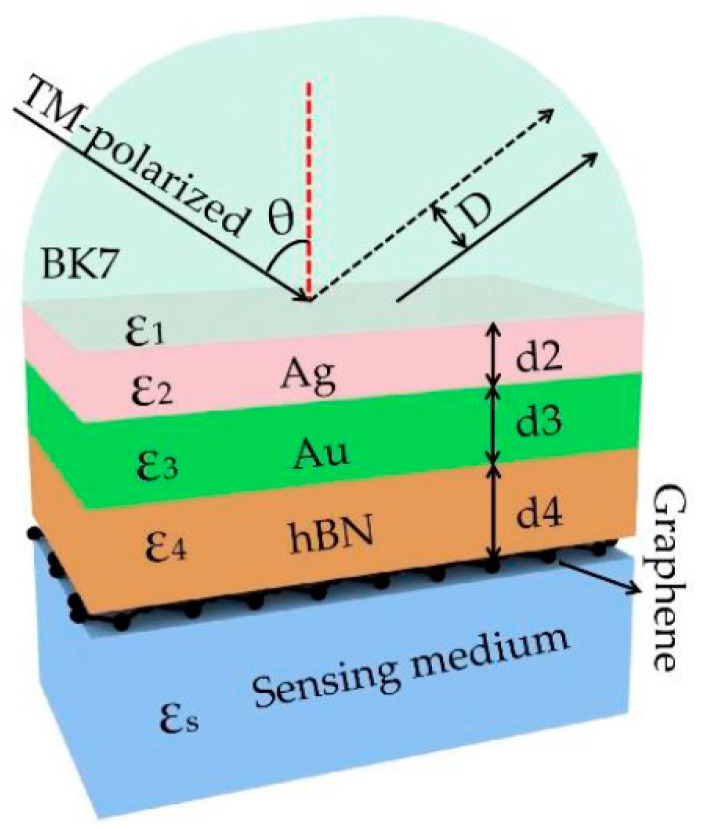
A bimetal structure based on graphene-hexagonal boron nitride (graphene-hBN) of schematic diagram.

The analysis structure of K-R is shown in Figure 1, which includes BK7 prism, Ag-Au bimetallic film, hBN, graphene, and sensing layer. When a transverse magnetic (TM)-polarized beam incident on BK7 prism, the evanescent-wave propagates along the prism metal interface and penetrates the metal layer. Once resonance occurs at a certain angle (resonance angle *θ*), the minimum reflectivity can be obtained. Thus, the phase mutation will be caused, and a large GH shift will be produced.

In this device, the dielectric constant of BK7 is given by the relationship [28]

(1) $$\varepsilon_{1}=\frac{1.03961212\lambda^{2}}{\lambda^{2}-0.00600069867}+\frac{0.231792344\lambda^{2}}{\lambda^{2}-0.0200179144}+\frac{1.01046945\lambda^{2}}{\lambda^{2}-103.560653}+1,$$

where *λ* is the incident wavelength and the unit is μm. According to the Drude-Lorentz model, the dielectric constant of the metal layer is [29]

(2) $$\varepsilon_{m}=1-\frac{\lambda^{2}\lambda_{c}}{\lambda_{p}^{2}{(\lambda }_{c}+i\lambda )},$$

Among them, *m* = 2,3. Here *λ_p_* = 1.4541 × 10^−7^ m and *λ_c_* = 1.7614 × 10^−5^ m correspond to the collision and plasma wavelengths of the Ag, respectively. For Au, *λ_p_* = 1.6826 × 10^−7^ m and *λ_c_* = 8.9324 × 10^−6^ m.

hBN has two infrared different reststrahlen active optical phonon modes in the infrared band. Its dielectric constant can be expressed as [26]

(3) $$\varepsilon_{u}{=\varepsilon }_{\infty ,u}\left(1+\frac{\omega_{\mathrm{LO},u}^{2}-\omega_{\mathrm{TO},u}^{2}}{\omega_{\mathrm{TO},u}^{2}-\omega^{2}-{i\omega \gamma }_{u}}\right),$$

In the formula, u=x,y represents the transverse direction (*a*,*b* axis direction of crystal face), u=z represents the *z* axis (*c* axis direction of crystal lattice). $\varepsilon_{\infty }$ and *γ* represent the high frequency dielectric constant and attenuation constant respectively. All data parameters here are taken from the literature [26].

The surface conductivity *σ* of graphene can be described in Kubo’s form. Without considering the external magnetic field, the surface conductivity *σ* of graphene can be expressed as the sum of in band conductivity and inter band conductivity [30]

(4) $$\sigma_{\mathrm{intra}}(\omega )=\frac{e^{2}K_{B}T}{{\pi h}^{2}}\frac{i}{{\omega +i\tau }_{g}^{-1}}\left[\frac{E_{F}}{K_{B}T}+2{\ln(e}^{-\frac{E_{F}}{K_{B}T}}+1)\right],$$

(5) $$\sigma_{\mathrm{inter}}(\omega )=\frac{{\mathrm{ie}}^{2}}{4{\pi h}^{2}}\ln\left[\frac{2E_{F}-{(\omega +i\tau }_{g}^{-1})h}{2E_{F}{+(\omega +i\tau }_{g}^{-1})h}\right],$$

Here, ω is the angular frequency of the incident light, e and h are the electron charge and the reduced Planck constant, respectively. $E_{F}$ and $K_{B}$ are Fermi energy and Boltzmann constants, respectively. *T* is the temperature in *K* and we set it to 300 K. Moreover, $\tau_{g}$ is the relaxation time of graphene.

The dielectric constant of the sensing layer can be expressed as

(6) $$\varepsilon_{s}{=n}_{s}{}^{2}=(1.33+\Delta n_{s}{)}^{2},$$

where $\Delta n_{s}$ is the amount of changes in refractive index of the sensing medium.

For multilayer structures, we can use the improved transfer matrix method to analyze the reflection phase ($\varphi_{r}$) and reflectivity ($R_{r}$). The transfer matrix of the composite structure can be expressed as [25]

(7) $${M=D}_{BK7\to \mathrm{Ag}}P_{\mathrm{Ag}}D_{\mathrm{Ag}\to \mathrm{Au}}P_{\mathrm{Au}}D_{\mathrm{Au}\to \mathrm{hBN}}P_{\mathrm{hBN}}D_{\mathrm{Sensing}},$$

Here, *D* represents the transfer matrix at the interface, and *P* is the propagation matrix in the medium. Then we can get $r=M_{21}/M_{11}$, where r is the reflected coefficient of the structure. At the same time, the corresponding reflectance $R_{r}{=|r|}^{2}$ and reflected phase $\varphi_{r}\left(\theta ,\omega \right)$ can be obtained by reflection coefficient. For the incident beam with sufficient waist, the GH shift of the reflected beam can be calculated by the steady-state phase method [12]

(8) DGH(θ,ω)=−λ2πdφrdθ=−λ2π1|r|2(Re(r)dIm(r)dθ−Im(r)dRe(r)dθ),

where *λ* is the corresponding incident wavelength.

The sensor of the GH shift interrogation mode analyzes the sensing performance by detecting the GH shift. Therefore, the sensitivity of the sensor can be defined as [16]

(9) $$S=\frac{\Delta D_{\mathrm{GH}}}{\Delta n_{s}},$$

Here, $\Delta D_{\mathrm{GH}}$ is defined as the amount of change in GH shift caused by changes in the refractive index of the sensing layer, and $\Delta n_{s}=0.005$.

## 3. Results and Discussion

Since the refractive index of the sensing layer of composite structure has a significant impact on the GH shift, the sensor based on GH shift interrogation mode can achieve the sensor performance by monitoring the GH shift signal. However, if the GH shift is too small, it may lead to errors in the monitoring results or not get better sensitivity, which will affect the application of refractive index sensor in real life. There are some important parameters of the composite structure that affect the GH shift, such as Fermi energy, the number of layers of graphene, thickness of hBN, and thickness of bimetal. The optimal sensitivity of the sensor can be obtained by finding a group of optimal structural parameters. Here, it is assumed that the incident wavelength of TM-polarized beam is *λ* = 10.176 μm. Also, the corresponding parameters are gradually optimized to improve the sensitivity of the structure.

**Figure 2:**
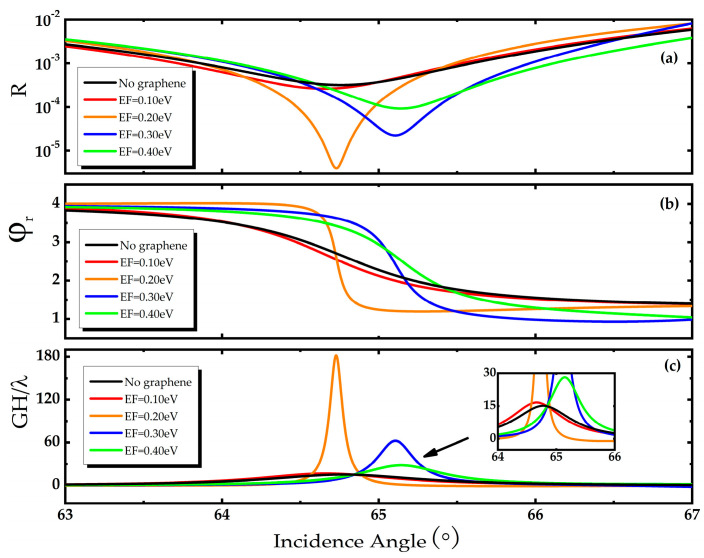
(**a**) Reflectance *R* as a function of incident angle at different Fermi energy, (**b**) Reflected phase $\varphi_{r}$ as a function of incident angle at different Fermi energy, (**c**) Goos–Hänchen (GH) shift as a function of incident angle at different Fermi energy, the inset is an enlarged view of the GH shift in the range of 64~66°, in which Ag thickness $d_{2}=15\;\mathrm{nm}$, Au thickness $d_{3}=20\;\mathrm{nm}$, hBN thickness $d_{4}=131\;\mathrm{nm}$, graphene layer *N* = 1.

First of all, the influence of Fermi energy on the reflectance, reflected phase and GH shift can be plotted and the corresponding curve is drawn in Figure 2. It can be seen from Figure 2a that there is an obvious dip of the reflectance near the resonance angle. The dip is caused by the excitation of surface plasmon polariton (SPP) on the surface of the metal medium when resonance occurs. Therefore, the power of the incident light is mostly absorbed and transferred to the SPP wave so that the reflectance curve drops sharply at the resonance angle. As the Fermi energy increases, the reflectance curve moves to higher angle. Then, the reflected phase curve also has an obvious mutation near the resonance angle. What’s more, the slopes of reflection phase curves are different near the resonance angle at different Fermi energy, which can be found in Figure 2b. It is worth noting that we know from Formula (8) that in order to obtain a large GH displacement, a large slope of the reflection phase is required. Therefore, by applying the gate voltage to tune the Fermi energy of graphene to alter it from 0.1 eV to 0.4 eV, it can be clearly seen from Figure 2b that the slope of the reflected phase first increases and then decreases. When *E_F_* = 0.2 eV, the reflected phase curve is the steepest. More importantly, two obvious features of GH shift can be found in Figure 2c. On the one hand, with the increase of Fermi energy, GH shift shows a tendency of increasing first and then decreasing. On the other hand, the corresponding GH shift is affected by reflection phases with negative slopes and shows positive values. At the same time, the reflected phase changes dramatically near the resonance angle, which makes the maximum GH shift value obtained. Especially, when the *E_F_* = 0.2 eV, the normalized GH shift of 182.09 λ can be obtained near the resonance angle. These phenomena further confirm the validity of regulating GH shift by voltage. This provides an effective way to enhance the GH shift flexibly and obtain the optimum sensitivity of the structure.

**Figure 3:**
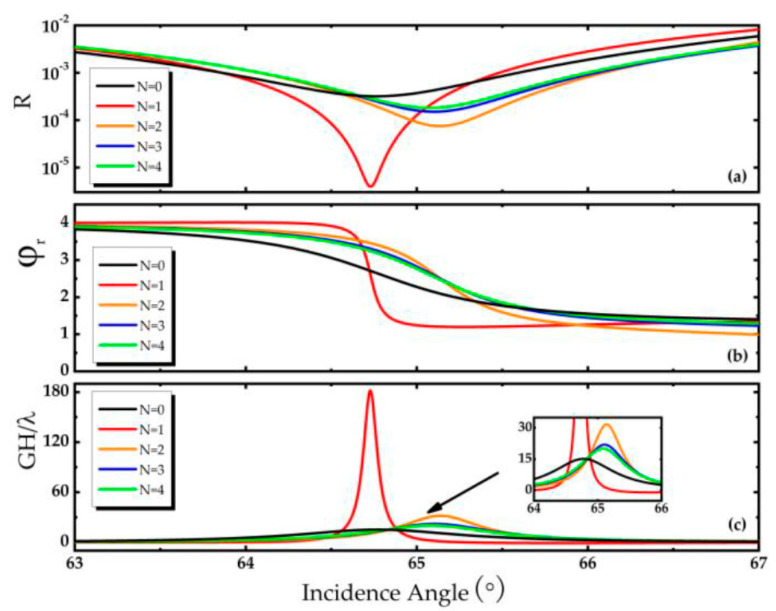
(**a**) Reflectance *R* as a function of incident angle at different layers of graphene, (**b**) Reflected phase $\varphi_{r}$ as a function of incident angle at different layers of graphene, (**c**) GH shift as a function of incident angle at different layers of graphene, in which Fermi energy *E_F_* = 0.2 eV, Ag thickness $d_{2}=15\;\mathrm{nm}$, Au thickness $d_{3}=20\;\mathrm{nm}$, hBN thickness $d_{4}=131\;\mathrm{nm}$.

Note that for graphene with a small number of layers (*N* < 6, where *N* is the number of layers), the optical conductivity of graphene with a small number of layers can be approximately expressed as *Nσ* if only considering the graphene sheets of each single atomic layer are non-contact [13]. Under this assumption, it is convenient to discuss the effect of the number of graphene layers on the GH shift, and the results are shown in Figure 3. On the whole, when the graphene layer is a single layer, the dip of reflectance is the deepest, and the reflection phase is the steepest, thus a large GH shift of 182.09 *λ* is obtained. Then, as the number of graphene layers increases, the reflectance in Figure 3a is enhanced and the dip becomes shallow. Also, the slope of the reflection phase is decreasing (see Figure 3b) which leads to a corresponding reduce of the GH shift. Fortunately, when graphene does not exist, there is a GH shift of about 15 *λ* in this composite structure. As mentioned above, if the sensitivity of the sensor is to be improved, the GH shift of the structure should be enhanced first, because this can make the detection of GH shift signal more accurate and convenient, and the sensitivity of the sensor can be improved correspondingly. It can be known from Figure 3 that the GH shift obtained when monolayer graphene is selected for calculation is the largest, so monolayer graphene is selected for the sensor sensitivity research in the following research.

**Figure 4:**
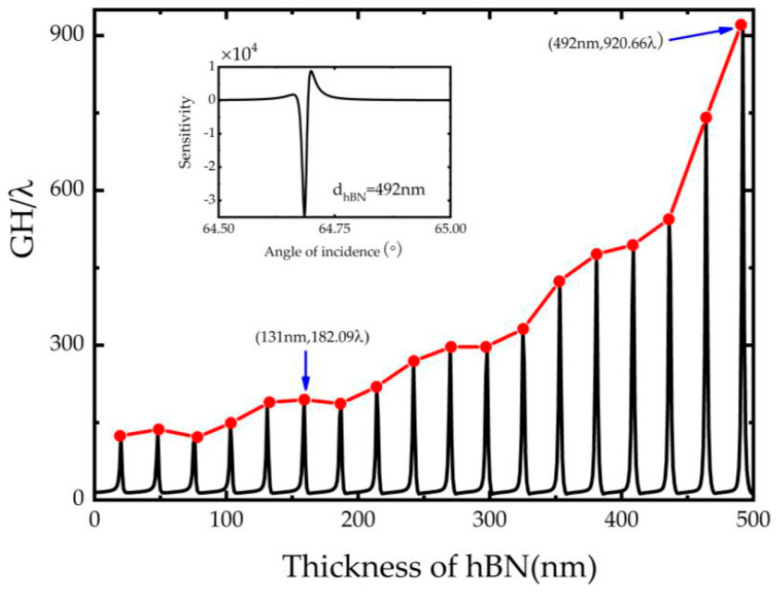
Dependence of GH shift on the thickness of hBN, in which Fermi energy *E_F_* = 0.2 eV, Ag thickness $d_{2}=15\;\mathrm{nm}$, Au thickness $d_{3}=20\;\mathrm{nm}$, graphene layer *N* = 1 and the inset is the sensitivity curve at $d_{3}=492\;\mathrm{nm}$.

At the same time, the thickness of hBN is also an important control parameter in increasing GH shift. Not only does it have Lorenz resonance characteristics in the infrared band, but it is also an important factor in the propagation matrix. Therefore, a curve is drawn in Figure 4, which enhances GH shift and improve the sensitivity of the structure by selecting the appropriate thickness. It can be found in the figure that the GH shift value increases initially then reduces, with the enlargement of hBN thickness (in a small range). In addition, the maximum GH shift keeps rising in a single period. When the hBN thickness is 492 nm, the GH shift reaches a normalized shift value of 920.66. Furthermore, in the illustration of Figure 4, the sensitivity curve is drawn with the thickness of hBN is 492 nm. Also, the sensitivity can be reached 3.18 × 10^4^ *λ/RIU* when the angle is 64.68°. However, the thickness of the material is too large, which is not conducive to the fabrication of ultra-thin refractive index sensor and may have some defects. So, in this paper, for the convenience of research, we choose the thickness of hBN as 131 nm, and the GH shift value obtained here is 182.09 *λ*.

We know that Ag is the most suitable material for providing high sensitivity, while Au has great advantages in excellent oxidation resistance and better chemical stability. Therefore, it is necessary to select the thickness of each layer reasonably for obtaining higher sensitivity and stability. In Table 1, we show the best sensitivity under different thickness of gold and silver. In general, when Au is constant, the sensitivity increases first and then decreases with the rising of Ag. Also, when Ag stays the same, the sensitivity increases initially then reduces with the enlargement of Au. More importantly, when the thickness of the gold layer is 20 nm and the thickness of the silver layer is 15 nm, the maximum sensitivity (2083 *λ/RIU*) can be obtained at *θ* = 64.72° by calculation. Therefore, the Au thickness of 20 nm and silver thickness of 15 nm are considered in the study of the composite structure, which is more convenient to obtain the ideal sensitivity.

**Figure 5:**
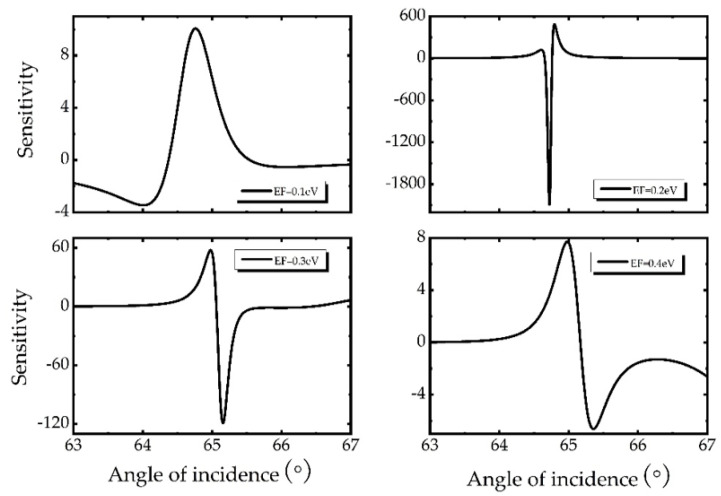
Dependence of sensitivity on Fermi energy, in which Ag thickness $d_{2}=15\;\mathrm{nm}$, Au thickness $d_{3}=20\;\mathrm{nm}$, hBN thickness $d_{4}=131\;\mathrm{nm}$, graphene layer *N* = 1.

In the previous discussion, we mentioned that Fermi energy has a great influence on GH shift and obtains a large GH shift (182.09 λ) at *E_F_* = 0.2 eV. Here, we will consider the sensitivity regulation of Fermi energy when the composite structure is applied to the sensor. In Figure 5, the sensitivity curve is plotted when the Fermi energy changes from 0.1 to 0.4 eV. With the increase of Fermi energy, the optimal sensitivity increases first and then decreases. Obviously, the trend of sensitivity with Fermi energy is consistent with the trend of GH. When the Fermi energy is 0.2 eV, the best sensitivity is obtained under the current parameter. It is further proved that enhancing GH shift value is an effective way to improve sensitivity.

**Figure 6:**
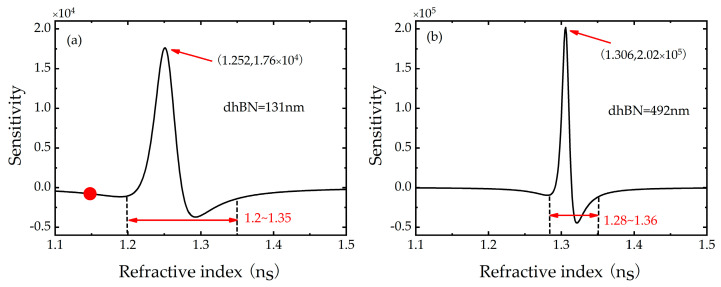
(**a**) Dependence of sensitivity of GH shift on refractive index ns of sensing layer when the hBN thickness is 131 nm, (**b**) Dependence of sensitivity of GH shift on refractive index ns of sensing layer when the hBN thickness is 492 nm, in which Fermi energy *E_F_* = 0.2 eV, Ag thickness $d_{2}=15\;\mathrm{nm}$, Au thickness $d_{3}=20\;\mathrm{nm}$, graphene layer *N* = 1.

After setting the structural parameters, the dependence of the sensitivity of the GH shift on the refractive index *n_s_* of the sensing layer will be discussed in Figure 6. When the hBN thickness is 131 nm and the Fermi energy is 0.2 eV, as shown in Figure 6a, the sensitivity curve of GH shift varies with refractive index *n_s_* of the sensing layer is plotted. When the refractive index changes between 1.2~1.35 *RIU*, the sensitivity curve changes drastically, and the sensitivity is higher in this range. Based on these two reasons, the variation of the refractive index of the sensing layer within this range is more convenient for detection in applications. At this time, the sensitivity can be achieved 1.76 × 10^4^ *λ/RIU*, while the refractive index is 1.252. As mentioned before, the GH shift can be enhanced by increasing the hBN thickness. Therefore, the sensitivity curve is plotted in Figure 6b, and the sensitivity can reach values as high as 2.02 × 10^5^ *λ/RIU* when the refractive index is 1.306. However, in Figure 6b, the range of sharp sensitivity varies is shifted to the right to 1.28–1.36, and the sensitivity detection range is slightly reduced. It can be concluded that the sensitivity can be improved by magnifying the thickness of hBN, but the best detection range will shift to a large refractive index and the detection range of high sensitivity will be narrowed correspondingly. Therefore, we can select the appropriate thickness of hBN according to the predicted refractive index range in practical application, and then tune the Fermi energy to advance the sensitivity. The refractive index sensor based on GH shift interrogation mode has the advantages of simple structure, high-sensitivity, and tunability, which are of great significance for the design of infrared band precise measurement or optical sensor.

## 4. Conclusions

In summary, we propose a bimetal structure based on graphene-hBN to enhance the GH shift in the infrared band and design a sensitivity-tunable refractive index sensor based on the GH shift. The results of numerical simulation show that when the Fermi energy is 0.2 eV, a large GH shift can be obtained in monolayer graphene. In addition, when the thickness of gold layer is 20 nm, silver layer is 15 nm, and the thickness of hBN is 131 nm, the sensitivity of the sensor can reach 1.76 × 10^4^ *λ/RIU*. Also, the sensitivity of the sensor can reach 2.02 × 10^5^ *λ/RIU* when the thickness of hBN is 492 nm, but the sensor can only be used to detect samples with high refractive index. Therefore, the thickness of hBN should be determined according to the refractive index of the sample. These results can provide a theoretical basis for the design of refractive index sensor with high sensitivity and accuracy in infrared band.

## Figures and Tables

**Table 1:** Optimum sensitivity (*λ/RIU*) for different gold and silver thicknesses.

Au/Ag(nm)	14	15	16	17	18	19
16	0.2747	0.3625	0.5110	0.8081	1.6794	29.3081
18	0.6258	1.0099	2.2402	777.6916	21.4966	4.7777
20	5.4571	2083	92.2965	7.7814	3.0161	2.7537
22	3.8497	8.1241	45.6784	437.8950	143.1114	21.2400
24	0.7618	0.7794	1.0741	1.3421	1.5868	1.9990
